# Efficacy of different biomechanical strategies for modulating force–time parameters of high-velocity low-amplitude manipulation of the thoracic spine: a randomized crossover experimental study

**DOI:** 10.1186/s12998-025-00585-0

**Published:** 2025-06-11

**Authors:** Grand Choi, Averie McGuinty, Nicole Meaghan Smith, Erinn McCreath Frangakis, David Starmer, Samuel J. Howarth, Simon Wang

**Affiliations:** 1 Division of Undergraduate and Graduate Education, Canadian Memorial Chiropractic College, 6100 Leslie Street, Toronto, ON M2H 3J1 Canada; 2 Department of Kinesiology, Faculty of Applied Health Sciences, University of Waterloo, 200 University Avenue, Waterloo, ON Canada; 3 Division of Research and Innovation, Canadian Memorial Chiropractic College, 6100 Leslie Street, Toronto, ON Canada

**Keywords:** Manual therapy, Spinal manipulation, Motor learning, Skill development, Force-sensing

## Abstract

**Background:**

Manual therapy, including high-velocity low-amplitude spinal manipulation (HVLA-SM), is a complex motor task performed by trained individuals. The ability to modulate the magnitude of applied forces is an attribute of proficiency that is challenging for providers and students. Adopting different biomechanical strategies may facilitate force modulation by practitioners performing HVLA-SM. This study evaluated the efficacy of different biomechanical strategies on force–time characteristics of prone thoracic HVLA-SM.

**Methods:**

A randomized crossover experimental design was used. Data were collected between October 2022 and May 2023 from chiropractic students at the Canadian Memorial Chiropractic College who performed HVLA-SM targeted to the thoracic spine of a prone-lying manikin using as much force as possible in each of six different strategies. Strategies (S1 to S6) were specifically developed to successively increase a person’s ability to produce force while performing HVLA-SM. Force–time parameters for the HVLA-SM trials were recorded. Peak force was the primary outcome of interest while preload force, load rate, and time to peak force were analyzed as secondary measures.

**Results:**

Data were collected from 97 participants (51 female). Peak force increased successively from S1 to S5 with moderate effects (− 0.45 ≤ effect size ≤ −0.72). There was no statistical difference in either peak force or load rate between S5 and S6. Load rate also did not statistically increase between S3 and S4 where different muscle groups were targeted to produce force. The strategy with the highest peak force (S6) also demonstrated the lowest preload force.

**Conclusions:**

Strategies used in this study effectively facilitated modulation of force–time characteristics of prone thoracic HVLA-SM. Thus, training approaches may consider introducing people to different biomechanical strategies to enhance HVLA-SM force modulation.

**Supplementary Information:**

The online version contains supplementary material available at 10.1186/s12998-025-00585-0.

## Background

Manual therapy, including high-velocity low-amplitude spinal manipulation (HVLA-SM) is a common guideline-recommended intervention for back and neck pain [1–4]. Performing the motor skill of HVLA-SM requires the provider to simultaneously coordinate movements of their extremities and trunk to control their total body center of mass while rapidly delivering forces to a recipient [5]. Thus, HVLA-SM represents a complex motor task and the development of proficiency in trainees has been the focus of many studies [6].

One skill for proficiency of people learning to perform manual therapy, identified by an expert consensus of manual therapy educators, is the ability to apply and control force [7]. Moreover, the Council on Chiropractic Education—Canada describes the ability for a chiropractor to “accurately control the speed, force, depth and distance of the adjustive thrust” as an example of therapeutic skill [8]. Modulating force–time characteristics such as the peak force during the impulse phase, impulse duration, and resulting loading rate have also been demonstrated to alter the neuromechanical response to HVLA-SM in animal preparations and humans [9–15]. Training of force modulation and the ability to demonstrate proficiency by achieving prespecified target forces have been studied for people performing HVLA-SM primarily using augmented feedback [6]; however, the biomechanical and motor control strategies to achieve force modulation has received comparatively less attention [16].

Modulation of HVLA-SM force–time characteristics can be achieved by the provider either consciously altering their physical effort, choosing a different biomechanical strategy or a combination of these two factors. Published studies either instruct participants to perform HVLA-SM with a qualitative descriptor of force magnitude (e.g., half-typical, typical, double-typical) or provide a prespecified force target (e.g., 400N, 600N) for the participant to achieve [17–20]. Details of participant body positioning for the procedures are limited. It is presumed that variation of force is achieved in these studies mainly by the increasing or decreasing one’s physical effort, since the predominant approach to training manual therapy skills favours mimicking the postures and movements of an instructor, instead of altering body position [21].

To our knowledge, there has only been one study to date that examined the effect of different performance strategies on the force–time characteristics of HVLA-SM [16]. Findings from this study collectively demonstrated the potential for different biomechanical strategies to influence a performer’s ability to control their delivery of forces during HVLA-SM; however, the investigators used a different clinician, ranging in body mass from approximately 65 kg to 127 kg, to perform each strategy and did not specify or standardize the clinician’s level of effort when performing the HVLA-SM procedures. Interestingly, the order of the peak applied forces from highest to lowest for the strategies was reversed when these data were normalized to the clinician’s mass. This emphasizes the point that it is difficult to ascertain the extent to which the observed differences in force–time characteristics are either a consequence of the three different strategies or reflect the performer.

The current study evaluated the efficacy of six different biomechanical strategies to modulate the force–time characteristics, primarily the total peak force, of maximal effort HVLA-SM directed to the thoracic spine of a manikin. Biomechanical strategies were defined by starting postural configurations of the performer and a set of motor cues provided by a trained instructor. Total peak force was hypothesized to follow graded increases from lower to higher as the initial position for the provider’s upper body moved away from the manikin (S2 and S3), with further increases as motor cues favoured force generation through involvement of different muscle groups (S4) and provider-generated momentum (e.g., body weight drop; S5 and S6).

## Methods

### Study design, setting, ethics and reporting

This study used a randomized crossover design and reporting of the study details was based on the 2010 Consolidated Standards of Reporting Trials extension to randomized crossover trials [22]. A crossover design was selected to expose the same subjects to different interventions (biomechanical strategies) to determine their impact on within-subject modulation of HVLA-SM force–time parameters. Reporting of HVLA-SM procedures was based on the Consensus on Interventions Reporting Criteria List for Spinal Manipulative Therapy [23].

Data collection occurred in the Force Sensing Table Technology (FSTT®) Simulation Lab at the Canadian Memorial Chiropractic College (CMCC). Study protocols were approved by the CMCC’s Research Ethics Board (REB #2207B02) and all participants provided written informed consent prior to enrollment. Participants received no compensation for participating.

### Participants and recruitment

The target population for this study comprised of students between the ages of 18 and 60 and who were enrolled in the Doctor of Chiropractic program at the CMCC. Potential participants were excluded based on any self-reported existing injury that restricted their ability to perform prone thoracic HVLA-SM. Participants were recruited between October 2022 and May 2023 through convenience sampling at the CMCC using email and social media announcements (X (formerly Twitter) and Facebook) as well as direct recruitment by researchers and research assistants.

### Randomization

#### Sequence generation

The order of the strategies was randomly determined for each participant by using simple randomization. A biostatistician who was not an investigator created the randomization sequence using a computer randomization program.

#### Allocation concealment and implementation

Allocation of randomized sequences of strategies was ensured using opaque envelopes which were only opened after enrolment and just prior to data collection by a research assistant. Participants were enrolled and assigned the sequence of interventions by research assistants (NS, AM, EMF).

### Interventions

All participants performed HVLA-SM using 6 different biomechanical strategies on a Human Analogue Manikin (HAM®) (Canadian Memorial Chiropractic College, Toronto, ON). The HAM® was chosen as a stand-in for live recipients due it its anthropometrically consistent soft tissue compliance and anatomical landmarks [24]. The HAM® was laid prone on an instrumented treatment table with the shoulders in line with the cephalad edge of the thoracic cushion. Each participant used a crossed bilateral hypothenar transverse hand contact for all HVLA-SM trials. Participants were instructed to provide a maximal-effort thrust (i.e., produce as much force as possible) in a posterior-to-anterior direction in the middle of the interscapular region of the manikin.

Prior to data collection for each strategy, a verbal description and a physical demonstration of the strategy were provided by an investigator (GC) with 5 years of experience teaching HVLA-SM technique and who was involved in developing the strategies that were evaluated in this study. Participants were allowed to perform 2 sub-maximal practice HVLA-SM trials followed by 3 maximal-effort trials for each strategy. Thirty seconds of rest was provided between each trial. If a strategy’s specific position or movement was not maintained or performed correctly, as determined by GC, or if the participant was dissatisfied with their performance when asked, the participant was asked to repeat the trial. The number of repeat attempts were recorded.

#### Description of biomechanical strategies

Six different strategies were utilized (Table 1). These strategies (S1-S6) were designed to theoretically affect a person’s ability to produce force while performing HVLA-SM by modulating aspects of leverage (e.g., elbow angle) and momentum (e.g., shifting of body mass). Specifically, the flexion angles of the provider’s elbows were successively decreased between S1, S2 and S3 with the same motor cue of generating an isolated elbow extension moment. Decreasing the elbow angle from S1 to S3 was also expected to reduce the moment arm length between the provider’s elbow, where the triceps brachii muscle attaches, and their hand contact on the manikin’s torso, which was standardized across the six strategies. A similar starting postural configuration was used for S3 and S4 with the main difference being a cue to apply force during the HVLA-SM using muscles of the shoulder and chest with greater force generating capacity in S4. Strategies 5 and 6 required participants to apply force using the same motor cue as S4 with additional force applied by modulating downward momentum of the participant’s total body center of mass.

**Table 1:** Illustrations of positioning for each strategy and accompanying instructions

Strategy	Instruction script	Position
S1	Bring your chest as close as possible to your contact hands, use only the extension of your elbows to create the thrust	
S2	Bring your chest a half arm length distance away from your contact hand, use only the extension of your elbows to create the thrust	
S3	Bring your chest to almost full arm length distance away from your contact hand, keeping the elbows slightly bent, use only the extension of your elbows to create the thrust	
S4	Bring your chest a full arm length distance away from your contact hand, elbows fully extended, use your shoulders [protraction] to create the thrust	
S5	Bring your chest a full arm length distance away from your contact hand, elbows fully extended, use your shoulders [protraction] AND a body weight drop to create the thrust	
		
S6	Start your body weight shifted to your back foot, elbows fully extended, then shift forward using momentum COMBINED with your shoulders [protraction] AND a body weight drop to create the thrust	

### Data collection and reduction

#### Instrumentation

Force–time data were collected for each HVLA-SM trial using the Force Sensing Table Technology (FSTT®) system (Canadian Memorial Chiropractic College, Toronto, ON, Canada). The FSTT® resembled a chiropractic treatment table and consisted of a custom steel frame with cushions and a 6 degree-of-freedom force plate (OR6-7, AMTI Inc., Watertown, MA, USA) embedded under the thoracic cushion. This device measured the three-dimensional reaction forces and moments at the interface between the table and the manikin during the HVLA-SM, which were used as surrogate representations of the loads applied by the clinician [25]. The force plate was calibrated by the manufacturer and was capable of recording forces up to 8900N along its X and Y axes and up to 17,800N along its Z-axis (i.e., perpendicular to the plane of the force plate’s surface) to within 0.5% of the applied force. Analog voltages from the force plate were digitally sampled at a rate of 2000 Hz using a ± 10 V range on a 16-bit analog-to-digital converter (USB-6259, National Instruments Inc., Austin, TX, USA). The FSTT®, which adds a cushion on top of the force plate, has demonstrated accuracy of dynamic forces in two studies to within an average of 2.2% across a range of forces from 89 to 222N and 4.3% at an undisclosed force magnitude [26, 27].

#### Data reduction

Discrete measures were either extracted or calculated from the recorded force–time profile of each HVLA-SM trial using custom software written in Matlab (The Mathworks Inc., Natick, MA, USA). Specifically, the preload force, total peak force and time to peak force were extracted while the load rate during the impulse phase of the HVLA-SM was calculated [25]. No changes were made to the data after extraction.

### Outcomes

The total peak force was the primary dependent measure given the study’s premise and that participants were instructed to elicit a maximal effort for each strategy. Preload force, time to peak force and the load rate were considered as secondary dependent measures.

### Blinding

Participants were not blinded to the order of the biomechanical strategies. Investigators who were responsible for data collection (GC, NS, AM and EMF) were also not blinded to the order of biomechanical strategies. Dependent measures were automatically determined by the FSTT® software and assigned to each participant’s unique code for deidentification of the data. The deidentified data were analyzed by an investigator (SH) who was not involved with either participant recruitment, data collection or data reduction; however, SH was not blinded to the strategies when analyzing the data. FSTT® measures were not visible to the participants.

### Statistical analysis

Sample means, standard deviations were calculated as descriptive statistics for demographic information.

Dependent measures (total peak force, preload force, time to peak force and load rate) were statistically evaluated using repeated measures analyses of variance based on a linear mixed effects model that considered a fixed effect of STRATEGY and random intercepts and slopes of STRATEGY within each participant [28]. Participant height, mass, sex and year of study were also entered into the model as covariates. Model assumptions were evaluated by visually inspecting plots of residuals and fitted values. Estimated marginal means and standard errors were calculated as descriptive statistics for the dependent measures.

Paired comparisons between each combination of STRATEGY were conducted as post hoc statistical tests. Effect size (ES) and the associated 95% confidence interval was also determined for each paired comparison. Strength of ES was interpreted using the 95% confidence interval and recommendations provided by Cohen (weak: $\left| {{\text{ES}}} \right| \le 0.2$, moderate: $0.2 < \left| {{\text{ES}}} \right| < 0.8$, strong: $\left| {{\text{ES}}} \right| \ge 0.8$) [29].

All statistical analyses were performed in R and used the *car*, *lme4*, *emmeans* and *predictmeans* packages [30–33]. Statistical significance was determined for p-values that were less than 0.001. The decision to use a more stringent criterion for statistical significance was to balance our methodological decision of not correcting for multiple comparisons.

### Sample size

The means and standard deviations of the peak force for S1 (mean = 566N, standard deviation = 105N) and S2 (mean = 625N, standard deviation = 52N) from Cambridge and colleagues were used to determine an effect size of 0.65 for a paired samples t-test [16]. This calculation assumed a correlation of 0.5 between measures for S1 and S2. Moreover, the comparison between S1 and S2 represented the smallest effect size of the three possible comparisons for peak force reported by Cambridge and colleagues [16]. The effect size of 0.65 was used alongside a Type I error rate of 0.1%, power of 80% and a two-tailed paired t-test to determine an estimated sample size of 46. However, we aimed to collect data from 100 participants given Harrell’s rule of thumb of 20 participants per variable for linear regression modeling and that our model included 5 variables [34].

## Results

### Participants

Data were collected from 97 participants. Demographic information for the 97 participants are presented in Table 2.

**Table 2:** Participant demographics. Standard deviations for height and mass are presented in parentheses

N	97
% Female	53
*Year of study*	
1	27
2	26
3	21
4	23
Height (m)	1.71 (0.10)
Mass (kg)	77 (14)

### Trial repeats

**Fig. 1:**
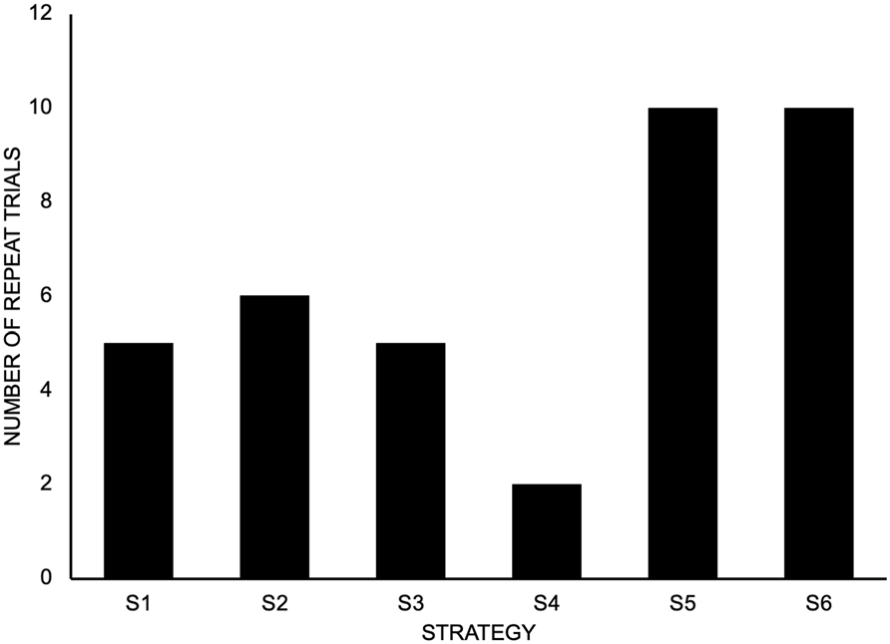
Number of repeat trials for each of the six strategies

A total of 38 trials were repeated because of either a break in form identified by GC or participant dissatisfaction with their performance. The highest number of repeated trials occurred for S5 and S6 (Fig. 1). Most participants (69%) did not incur a repeated trial. The largest number of repeated trials for any participant was 2 (8% of participants).

### Assumptions of statistical analyses

Plots of the residual and fitted values are provided in the Supplementary Materials. Total peak force, preload force and load rate met the statistical modelling assumptions. Time to peak force did not satisfy the homogeneity of variances assumption.

The model fit for time to peak force improved by using a Gamma distribution and z-scaled values for participant height and mass; however, there remained evidence that the homogeneity of variances assumption was violated by this updated model. Inspection of the output from the original model and the updated model revealed no difference in interpretation. We decided to use output from the model fitted using the Gamma distribution, despite not satisfying the homogeneity of variances assumption, given that time to peak was a secondary outcome for this study. All presented data for time to peak were back-transformed from log-scaled units to the original scale.

### Effects of biomechanical strategies on force–time characteristics

Estimated marginal means, standard errors, effect sizes and their confidence intervals are reported for total peak force, load rate, time to peak force and preload force in Tables 3, 4, 5 and 6.

**Table 3:**
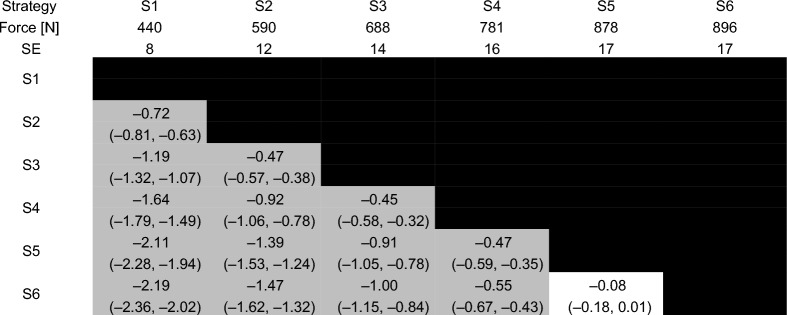
Total peak force for each of the six strategies

Estimated marginal means and standard errors (SE) are reported in the second and third rows. Effect sizes are reported in each cell with the 95% confidence interval for each effect size in parentheses. Effect sizes for comparisons between each pair of strategies with a p-value that was less than 0.001 are reported in the gray shaded cells

**Table 4:**
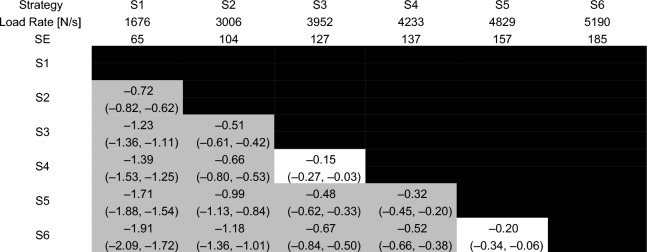
Load rate for each of the six strategies

Estimated marginal means and standard errors (SE) are reported in the second and third rows. Effect sizes are reported in each cell with the 95% confidence interval for each effect size in parentheses. Effect sizes for comparisons between each pair of strategies with a p-value that was less than 0.001 are reported in the gray shaded cells

**Table 5:**
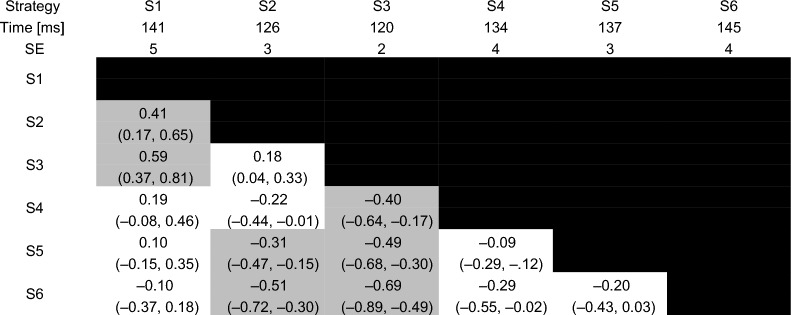
Time to peak force for each of the six strategies

Back-transformed estimated marginal means and standard errors (SE) from the model fitted using a Gamma distribution are reported in the second and third rows. Effect sizes are reported in each cell with the 95% confidence interval for each effect size in parentheses. Effect sizes for comparisons between each pair of strategies with a p-value that was less than 0.001 are reported in the gray shaded cells

**Table 6:**
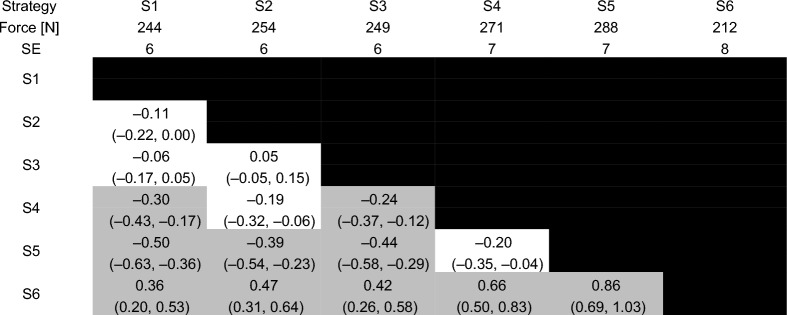
Preload force for each of the six strategies

Estimated marginal means and standard errors (SE) are reported in the second and third rows. Effect sizes are reported in each cell with the 95% confidence interval for each effect size in parentheses. Effect sizes for comparisons between each pair of strategies with a p-value that was less than 0.001 are reported in the gray shaded cells

Total peak force was statistically greater between successive strategies from S1 to S5 (i.e., S1 vs. S2, S2 vs. S3, S3 vs. S4, S4 vs. S5) with moderate effects (*p* ≤ 0.001, −0.72 ≤ ES ≤ −0.45). The only exception was between S5 and S6, which did not produce a statistically significant difference in total peak force (*p* = 0.076, ES = −0.08 [− 0.18, 0.01]).

Load rate increased with moderate effects between S1 and S2 (*p* < 0.001, ES = −0.66 [− 0.76, −0.57]), S2 and S3 (*p* < 0.001, ES = −0.99 [− 1.18, −0.81]) and S4 and S5 (*p* < 0.001, ES = −0.34 [− 0.46, −0.21]) strategies. There was no difference in load rate between S3 and S4 (*p* = 0.015, ES = −0.24 [− 0.35, −0.12]) and between S5 and S6 (*p* = 0.007, ES = −0.24 [− 0.36, −0.11]).

Time to peak force was lowest for S3 with statistically significant differences compared to S1, S4, S5 and S6 (*p* < 0.001). These differences represented weak-to-moderate effects.

Preload force was lowest for S6 with moderate-to-strong effects (*p* < 0.001, 0.36 ≤ ES ≤ 0.86). The highest preload force occurred for S5, which was statistically different from the other strategies (*p* < 0.001, −0.86 ≤ ES ≤ −0.39) except S4 (*p* = 0.013, ES = −0.20 [− 0.35, −0.04]). There were no statistically significant differences in preload force between S1, S2 and S3 (*p* ≥ 0.059, −0.11 ≤ ES ≤ −0.05).

## Discussion

The current study sought to evaluate the efficacy of six different biomechanical strategies for modulating force–time characteristics of HVLA-SM applied to the thoracic spine of a manikin. Statistically distinct total peak forces during maximal effort HVLA-SM were quantified for five of the strategies, which was consistent with the study’s hypothesis; however, total peak force for S6 was not statistically different from S5. The different strategies also influenced preload, load rate and the time to peak force. Findings from this study may have an impact on approaches to training of HVLA-SM procedures that has favoured mimicking a single postural configuration and movement strategy of an instructor.

To our knowledge, there has only been one other study to have reported force–time characteristics for different strategies of the same HVLA-SM procedure [16]. The positions of S2 and S5 from the current investigation were the most like the first and second strategies described by Cambridge and colleagues [16]. The mean peak force of S2 (590N) was very similar to the first Cambridge strategy (566N); however, mean peak force for S5 (878N) was larger than the second Cambridge strategy (625N). One contributor to this difference could have been that the instructions for S5 included the use of a bodyweight drop combined with protraction of the shoulders, whereas Cambridge only used a “lower body release” (bodyweight drop) without additional shoulder protraction. The absence of a “chest bump” in strategies used in the current investigation meant that there was no reasonable comparator for the third strategy used by Cambridge and colleagues [16].

Any comparison of findings between the current investigation and that of Cambridge and colleagues [16] must also consider the other methodological differences between the studies. Notably, the current study used a repeated measures design where each participant performed all six HVLA-SM strategies. Participants in the current study were also instructed to perform each HVLA-SM with a maximal effort as a way of standardizing effort across each strategy. Conversely, Cambridge and colleagues used a different clinician for each strategy, and they did not specify instructions given to the clinicians regarding their effort for performing the HVLA-SM procedures. Mass of the HVLA-SM provider could substantially influence the relationship between strategies and the total peak force during the impulse phase in both studies. Cambridge and colleagues also reported the total peak force scaled to the clinician’s weight to address inter-clinician differences in body mass. Interestingly, the scaled total peak force decreased from the first to the second strategies. This does not conform to the biomechanical principles upon which S2 and S5, the two comparable strategies to the first two strategies of Cambridge and colleagues, were developed and the data presented in the current study. It is possible that this difference is related to a combination of the explicit or implicit instructions that were provided to clinicians/providers in both studies and each study’s design.

Load rate, the time to peak force and preload force were quantified as secondary measures for this investigation. Mostly strong effects of differences between strategies were observed for load rate, which is defined as the change of force over a window of time. The strong effects of load rate differences between strategies were likely due to the increase in peak force achieved in a similar duration.

Preload was lowest for S6, which could be related to differences in the shape of the HVLA-SM force–time profile prior to the impulse phase. The preload phase for a posterior-to-anterior thoracic HVLA-SM procedure is frequently characterized by a convex curvature of the force–time profile, which achieves a local maximum before the impulse is initiated [35, 36]. The local maximum of the force–time profile represents a momentary pause as the provider transitions from the deliberate build-up of force during the preload phase to the rapid application of force during the impulse phase. Force–time profiles of HVLA-SM procedures can also either exhibit a transition from the convex shape of the preload phase to a concave shape at the start of the impulse phase without the occurrence of a local maximum, or the force–time profile can resemble an exponential relationship that is entirely concave. Combining the instructions for providers to start with their weight on their back foot and to perform the HVLA-SM procedure using a continuous forward and downward movement of their total body center of mass with the objective of applying maximum force likely meant that the momentary pause at the transition from the preload to the impulse phase did not occur for S6. In this case, the lower preload force that was reported for S6 is likely a result of a higher loading rate prior to the impulse phase of the HVLA-SM procedures, which crossed the software program’s threshold for identifying the onset of the impulse phase at a force that was lower than the preload force in the other strategies. Thus, the algorithm that automatically identified key points of the HVLA-SM procedures may have defined the same points for the preload and start of the impulse phase in S6. This was not quantified for the current investigation. Cambridge and colleagues [16] reported a similar relationship for their third strategy, which had the highest peak force with the lowest preload force.

Findings from the current study must be interpreted with consideration of its main (de)limitations. First, participants performed the HVLA-SM for all six strategies on a manikin and using maximal effort. These were methodological decisions to standardize aspects of the HVLA-SM across participants and strategies since the primary objective of the current investigation was to evaluate the efficacy of different biomechanical strategies for modulating the force–time characteristics. Participants in our study also performed all HVLA-SM trials with a crossed bilateral hypothenar-transverse hand contact. Previous work indicating that force production during HVLA-SM is impacted by different hand contacts may delimit applicability of our findings and is something for future research to consider [37]. Nonetheless, the internal validity gained by these methodological decisions is offset by a decrease in direct application to clinical practice where physical characteristics vary between patients, and clinicians purportedly would determine an appropriate amount of force to use instead of a maximal effort. It is important to emphasize that the findings of this study lend support to the use of different biomechanical strategies for achieving force modulation; however, the methodological decision to have participants perform maximal effort HVLA-SM precludes inferences regarding force control. Familiarity and novelty between the strategies is another limitation that may have affected participants’ performance of the HVLA-SM procedures. Participants were familiar with S3, S4 and S5 through their educational training. Conversely, S1, S2 and S6 were novel to participants. The two practice trials with submaximal effort for each strategy may have been insufficient for participants to adequately determine an approach to maximize force production in unfamiliar strategies. Finally, the data analysis was conducted by an investigator (SH) who was not blinded to the strategies when analyzing the data. Bias was effectively mitigated by this investigator not being involved with any aspects of the methodology that could impact dependent measures prior to analysis (i.e., participant recruitment, data collection, data reduction), and by not making changes to the data after extraction.

## Conclusions

This study demonstrated the efficacy of using different strategies, developed on biomechanical principles, for modulating force–time characteristics of HVLA-SM. Thus, training approaches may consider introducing people to different biomechanical strategies to enhance HVLA-SM force modulation. The current study took the approach of prescribing HVLA-SM strategies that theoretically would demonstrate force modulation when participants were asked to provide a maximal effort. Future work may consider evaluating the biomechanical strategies adopted by providers when tasked with modulating force.

## Supplementary Information

**Additional file 1.**

## Data Availability

The datasets generated and analyzed during the current study are not publicly available due to ethics restrictions but are available from the corresponding author on reasonable request.

## Abbreviations

CMCC: Canadian Memorial Chiropractic College
ES: Effect size
FSTT®: Force Sensing Table Technology
HAM®: Human Analogue Manikin
HVLA-SM: High-velocity low-amplitude spinal manipulation
S1, S2,…: Strategy 1, Strategy 2,…
