# Supplementary material for: Efficacy of different biomechanical strategies for modulating force–time parameters of high-velocity low-amplitude manipulation of the thoracic spine: a randomized crossover experimental study
Source: Chiropr Man Therap. 2025 Jun 11;33:25. doi: 10.1186/s12998-025-00585-0 (PMC12160424; doi:10.1186/s12998-025-00585-0)

**Supplementary Material –** Plots used to evaluate assumptions of statistical models for each dependent measure.

**(Primary) Total Peak Force**


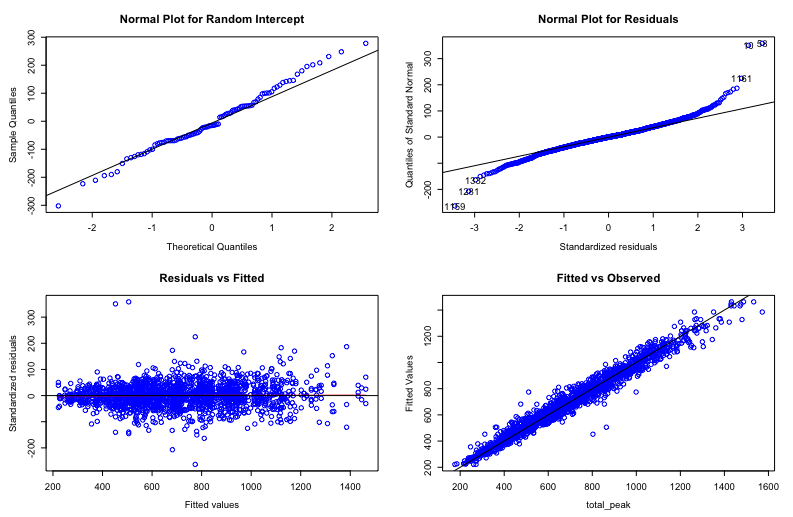


**Preload Force**


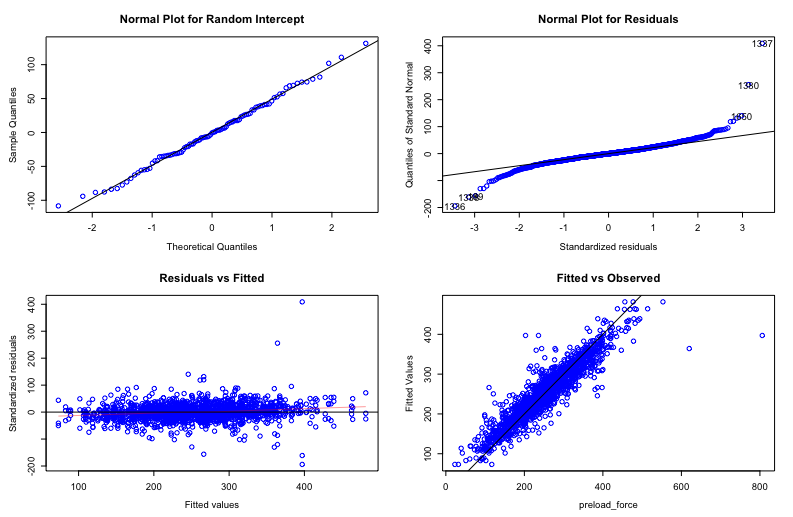


**Time to Peak Force (Normal Distribution)**


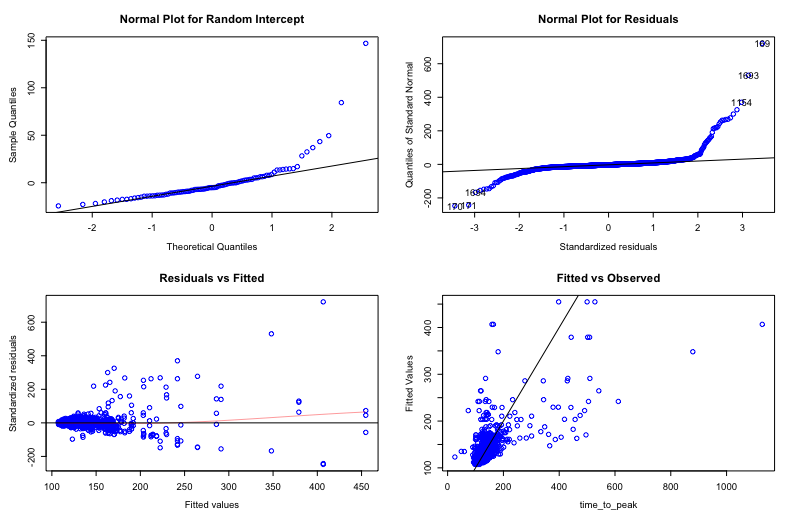


**Time to Peak Force (Gamma Scaled Distribution)**


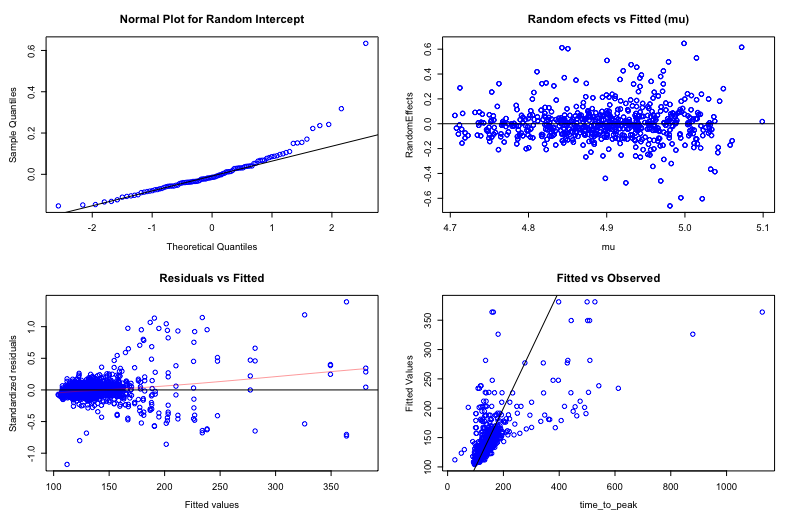


**Load Rate**


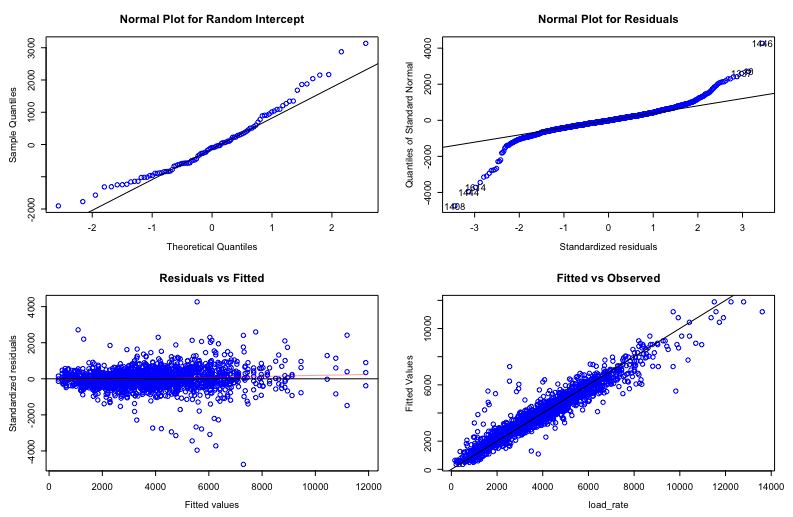

Supplement: Supplementary file 1 — Additional file 1. [file 12998_2025_585_MOESM1_ESM.docx]
